# Energy and the food system

**DOI:** 10.1098/rstb.2010.0172

**Published:** 2010-09-27

**Authors:** Jeremy Woods, Adrian Williams, John K. Hughes, Mairi Black, Richard Murphy

**Affiliations:** 1 Porter Alliance, Centre for Environmental Policy, Imperial College London, London SW7 2AZ, UK; 2 Porter Alliance, Department of Biology, Imperial College London, London SW7 2AZ, UK; 3 Natural Resources Management Centre, Department of Natural Resources, University of Cranfield, Bedford MK43 0AL, UK; 4 Agri-Environment and Land Use Strategy, Food and Environment Research Agency, Sand Hutton, York YO41 1LZ, UK

**Keywords:** energy in agriculture, fossil energy, agricultural greenhouse gas emissions, land use, agroforestry, policy

## Abstract

Modern agriculture is heavily dependent on fossil resources. Both direct energy use for crop management and indirect energy use for fertilizers, pesticides and machinery production have contributed to the major increases in food production seen since the 1960s. However, the relationship between energy inputs and yields is not linear. Low-energy inputs can lead to lower yields and perversely to higher energy demands per tonne of harvested product. At the other extreme, increasing energy inputs can lead to ever-smaller yield gains. Although fossil fuels remain the dominant source of energy for agriculture, the mix of fuels used differs owing to the different fertilization and cultivation requirements of individual crops. Nitrogen fertilizer production uses large amounts of natural gas and some coal, and can account for more than 50 per cent of total energy use in commercial agriculture. Oil accounts for between 30 and 75 per cent of energy inputs of UK agriculture, depending on the cropping system. While agriculture remains dependent on fossil sources of energy, food prices will couple to fossil energy prices and food production will remain a significant contributor to anthropogenic greenhouse gas emissions. Technological developments, changes in crop management, and renewable energy will all play important roles in increasing the energy efficiency of agriculture and reducing its reliance of fossil resources.

## 1. Introduction

The future for farming and agriculture holds many challenges, not least the continued efforts to optimize energy inputs and reduce greenhouse gas (GHG) emissions. This needs to be set against the urgent and growing need to improve yields to meet the anticipated requirements to provide food, feed, fuel, chemicals and materials for the growing global population. These challenges are and will increasingly be influenced by the availability and price of oil, natural gas and coal, as well as by policies set to meet carbon emissions targets and other sustainability requirements. This paper aims to investigate the impact of energy inputs on agricultural systems to the farm gate, for the production of key commodities. It has a strong UK focus but draws conclusions where possible from an international perspective.

The paper reviews the impact of current and future agricultural production on climate change and policies associated with reducing GHG emissions and finally considers options for reducing the dependency of agriculture on energy by considering alternatives, including the optimization and integration of land use for multi-purpose outcomes.

## 2. Energy use for food production

**Figure 1.**
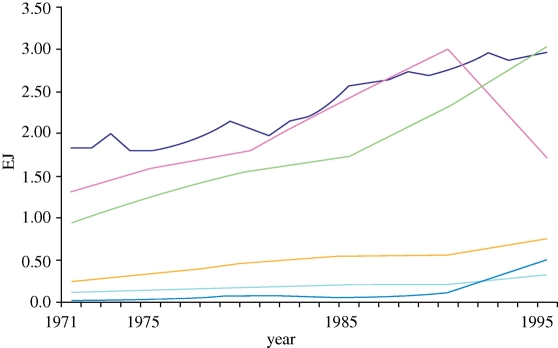
Primary energy use in agriculture, 1970–1995. Source: IPCC (2001). Light blue line, total fertilizers per ha cropland; brown line, cereal yield; purple line, total area equipped for irrigation; green line, tractors per ha; dark blue line, agricultural labour per ha cropland.

The 3rd Assessment report of the Intergovernmental Panel on Climate Change (IPCC 2001) estimated that by 1995, agriculture accounted for about 3 per cent (9 EJ) of global energy consumption, but more than 20 per cent of global GHG emissions. Figure 1 highlights the trend of increasing energy inputs to agriculture since 1971 and shows the high degree of variability both between regions and over time, for example, the collapse in energy inputs in the former Union of Soviet Socialist Republic (USSR) after the fall of the iron curtain in 1989.

Substantial areas of agricultural land also came out of production as these (former USSR) farms became exposed to global competition with governments unable to continue subsidizing production.

The links between agricultural energy inputs, yields, economic returns, land requirements and land-use change (LUC) needs further research. However, LUC has major implications for GHG emissions and carbon stocks, particularly where forest land is cleared or where previously arable land is allowed to revert to forest. These issues are discussed briefly in the ‘indirect emissions’ section below but are not a major focus in this paper.

**Figure 2.**
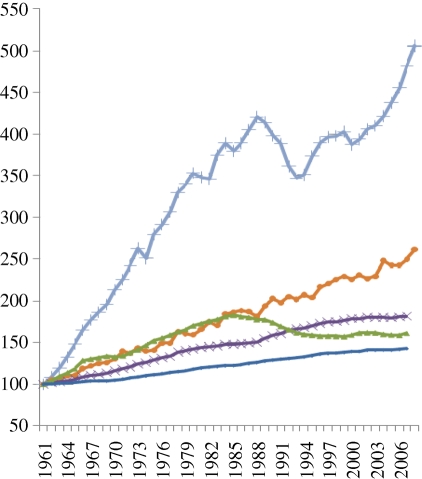
Global trends in the intensification of crop production (index 1961–2002/2005). Source: updated from Hazel & Woods (2008) based on FAOSTAT 2010. Dark blue line, industralized countries; pink line, economic in transition; green line, developing countries in Asia–Pacific; sky blue line, Africa; yellow line, Latin America; cyan line, Middle East.

If energy consumption by agriculture continued to grow at the annual rate outlined by the IPCC for 1995 (IPCC 2001), total energy inputs into agriculture would have exceeded 10 EJ in 2005, equivalent to a share of about 2 per cent of global primary energy consumption. Therefore, agricultural demand for fossil energy, while growing, represents a relatively insignificant and shrinking share of the overall fossil energy supply market. On the other hand, as yields and the inputs needed to support those yields increase, agriculture is becoming more dependent on fossil fuels, either directly for tillage and crop management or through the application of energy-intensive inputs e.g. nitrogen fertilizer and pesticides. Furthermore, the embodied energy in tractors, buildings and other infrastructure necessary to support agriculture and food supplies is likely to continue to grow as developing agricultural producers invest in the infrastructure needed to increase yields and become competitive in the global food commodity markets as outlined in figure 2 (IPCC 2001).

Embodied energy is all the energy used in the creation of a product. In the life cycle assessment (LCA) described subsequently, it is assumed that the long-term phosphorous (P) and potassium (K) requirements of all crops must be met.

**Figure 3.**
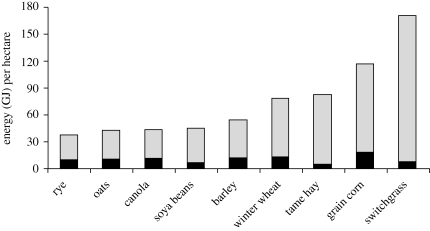
Solar energy collection in harvested component of crops and fossil fuel energy requirements of Canadian (Ontario) crop production, in Giga-Joules (GJ) per hectare. Source: Samson *et al*. (2005). Grey bars, energy content of crop per hectare less fossil-fuel energy consumption; black bars, fossil energy consumption per hectare production.

Fossil energy inputs into agriculture have generally been outweighed by yield improvements that deliver positive energy ratios (energy out: energy (fossil) inputs) ‘i.e. the energy content of the harvested crop is greater than the fossil energy used to produce the crop*,’* as highlighted by Samson *et al*. (2005), in figure 3. Future technologies that will allow both the higher value starch, oils and/or protein fractions to be harvested along with the lower value lignocellulosic fractions will improve the energy ratios and apparent nutrient use efficiencies of conventional food crops in comparison to dedicated biomass crops, such as switchgrass, as shown. However, over the full life cycle of a crop, particularly where energy-intensive drying and processing are required, in some cases more fossil energy can be used than is contained in the final product. A detailed assessment of the energy inputs and GHG emissions from UK agriculture in food production systems follows. While much of this assessment is specific to the UK, the heterogeneity in inputs, energy carriers, energy intensities and resulting GHG emissions for different crops is considered a conservative representation of commercial agriculture globally.

### (a) Contemporary UK agriculture

This section covers the main commodities produced in the UK and is from the perspective of LCA, which is a standard method for assessing the ‘cradle to grave’ environmental impacts of a product or process. The detailed breakdown that follows comes from the work of Cranfield University and is reported in various outputs (Williams *et al*. 2006, 2009; Audsley *et al*. 2010). The work was parameterized for England and Wales, although much applies in other parts of the UK. The original study included three field crops (bread wheat, oilseed rape and potatoes), four meats (beef, poultry, pork and lamb), milk and eggs. Tomatoes were included as the main protected crop. Apples and strawberries were analysed in a later study, together with overseas production of apples, potatoes, tomatoes, strawberries, lamb, beef and poultry meat. Primary production up to the farm gate was included in all these studies, although in Williams *et al*. (2009), the endpoint was the regional distribution centre. Studies have been carried out by various authors as reported by Pretty *et al*. (2005), who make an analysis of transport costs from farm to plate or ‘food’ miles, and substantial gains are possible in energy efficiency and waste reduction beyond the farm gate. However, this paper has focused on reviewing energy inputs for production to the farm gate.

With LCA, all energy use is traced back to resources in the ground, so that overheads of extraction and distribution are included in reported energy figures. All inputs are considered, so that the embodied energies in fertilizer, machinery, buildings and pesticides are included along with the direct energy of diesel and other fuels (also known as energy carriers). Estimates for the energy inputs into animal production include inputs for the production of all feed crops e.g. UK feed wheat, UK field beans, American soya and forage (grazed grass and conserved grass or maize) and for feed processing and distribution. All breeding overheads are also included, so that the final values represent the totality of energy used per commodity.

One of the challenges of these analyses is how to allocate burdens when crops are multi-functional. Oilseed rape is grown primarily for oil, but a useful meal is also produced as the result of oil extraction, which can be used as an animal feed. It is common practice with products of disparate properties to allocate burdens by economic value, rather than simply by weight or energy content, and this approach has been used here.

#### (i) Arable crops

Energy inputs to produce the UK's main crops (table 1) range from 1 to 6 GJ t^−1^. However, each agricultural product has very different properties and uses, making comparisons using a single metric problematic. Farming systems employed to grow crops will also influence outcomes for energy input, GHG emissions and potentially yield. Making comparisons between conventional and organic farming systems often leads to the general conclusion that organic provides a more energy-efficient system than conventional farming, but fossil energy input reduction has to be balanced against human energy inputs, which are often higher for organic systems (Zeisemer 2007). Comparisons of conventional farming and integrated arable farming systems (IAFS) have been reported by Bailey *et al*. (2003), suggesting that IAFS has lower energy inputs per hectare, but that this is balanced out by reduced yield reported for this set of results.

**Table 1.** Primary energy used in arable crop production (GJ t^−1^). All values are for England and Wales, except soya, sugarcane and maize. Source: based on Williams *et al*. (2006).

	primary energy used, GJ t^−1^		
	non-organic	organic^a^	national ‘basket’^b^
bread wheat	2.52	2.15	
oilseed rape	5.32	6.00^c^	
potatoes (national commodity level)			1.39
potatoes main crop	1.46	1.48	
potatoes 1st earlies	1.40	1.25	
potatoes 2nd earlies	0.79	0.75	
feed wheat	2.32	2.08	
winter barley	2.43	2.33	
spring barley	2.27	2.64	
field beans	2.51	2.44	
soya beans (US)	3.67	3.23	
sugarcane (Brazil)^d^	0.21		
maize (US)^e^	2.41		

^a^Based on long term yields obtainable from stockless rotations.

^b^‘National basket’ used to provide national average energy input for ‘average’ potato.

^c^Very little grown currently.

^d^Per tonne of harvested sugarcane delivered to the mill, 2005/2006: sample of 44 mills (100 Mt cane per season), all in the centre-south Brazil; data as reported by Macedo (2008).

^e^Per tonne of harvested maize grain. Derived from Farrell (2006).

Oilseed rape stands out as being the highest energy consumer per tonne of product, resulting from relatively low yields and high fertilizer requirements, but the grain is more energy-rich than cereals or legumes. Bread wheat receives more fertilizer than feed wheat, in order to obtain the high protein concentrations that are required for bread-making, and so takes more energy than feed wheat. Although field beans require no nitrogen (N) fertilizer, they have much lower yields than wheat and more diesel is used per tonne of beans produced.

**Figure 4.**
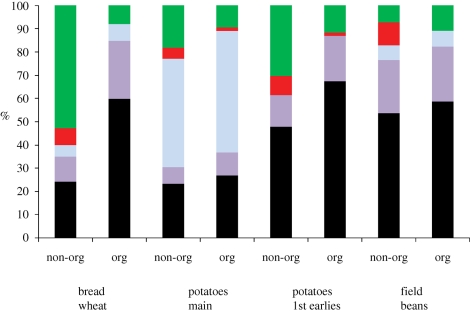
Breakdown of energy used in major domestic crop production. Source: Williams *et al.* (2009). Green bars, fertilizer manufacture; red bars, pesticide manufacture; blue bars, post harvest; purple bars, machinery manufacture; black bars, field diesel.

Cereals tend to follow the same pattern in terms of energy inputs and wheat is used here as a proxy for cereals in general (figure 4). UK wheat also has a similar energy input intensity to US maize production as shown in table 1. In non-organic bread wheat production, over half of the energy used is in fertilization and about 90 per cent of that energy is in N, typically ammonium nitrate (AN) and urea. Bread wheat is unusual in that urea is applied relatively late in the growth season, as a foliar feed. Direct field energy is just under a quarter of the input. Post-harvest energy inputs are mainly for grain drying and cooling, which were calculated here on a long-term basis: this clearly varies yearly according to climatic conditions. Pesticide manufacture accounts for less than 10 per cent of energy input, but a lack of modern data leads to higher degrees of uncertainty about the impacts of pesticide use, with the most recent publicly available analysis by Green (1987). In contrast, organic production uses more diesel per unit production, owing to lower yields and the obligation to use the plough, coupled with extra cultivations for weed and pest control.

Potato cropping is energy-intensive compared with cereals and legumes. For example, the energy used in storage is much larger than other crops: potatoes are kept cool and a proportion is maintained over the year. This is in contrast to traditional low-energy clamping systems, in which losses are much higher, but the supply season shorter. Early potatoes are generally not stored on farms, so energy requirements for field operations incur a major fraction of total energy inputs, which also include irrigation inputs as well as the high energy costs of planting, cultivating and harvesting. However, because potatoes are high-yielding crops, they have low-energy input requirement per tonne harvested. If calculated per tonne of harvested dry matter, because the harvested biomass is 80 per cent water for potatoes, compared with 15–20% for wheat grain, for example, potatoes would have a higher energy intensity factor.

Sugarcane production under Brazilian conditions and management is also high-yielding and has a high water content (70% moisture content) when harvested. The relatively low-energy inputs needed for the production of this semi-perennial crop and lower moisture content compared with potatoes mean that when accounting for energy intensity on a dry weight basis, sugarcane would have a lower energy intensity than UK wheat. Even when processed to ethanol and/or crystalline sugar, because of the use of residual biomass arising from sugar extraction to provide power and heat, fossil energy inputs are minimized.

**Figure 5.**
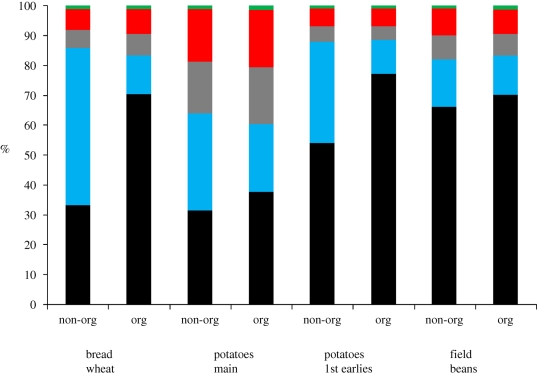
Distribution of energy carriers used in field crop production. Source: Williams *et al.* (2009). Green bars, renewable %; red bars, nuclear %; grey bars, coal %; blue bars, natural gas %; black bars, crude oil %.

The types of energy used vary between crops and production systems (figure 5), and also location. In the UK, as with most of Europe, nitrogen fertilizer production uses mainly natural gas. However, according to He *et al*. (2009), in China, coal currently provides about 80 per cent of the energy inputs into nitrogen fertilizer production, rising from 71 per cent in 2004. Diesel comes from crude oil. Electricity used either directly (e.g. cooling grain) or indirectly in machinery manufacture, also uses coal, nuclear and some renewables. The dominant energy carrier in non-organic wheat production is thus natural gas, but it is crude oil in organic wheat production and in China it would be coal. The embodied energy in machinery is an overhead of about 40 per cent of the energy used in diesel, reflecting the high wear environment of cultivating and harvesting, as well as continually high power demand on engines, compared with road transport.

**Figure 6.**
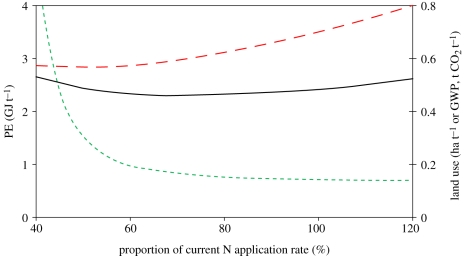
Effects of changing N supply on bread wheat using the Cranfield model. PE, Primary Energy; GWP, Global Warming Potential. Source: Williams *et al.* (2006). Black line, PE; red long dashed line, GWP; green long dashed line, land use.

Although fertilizer manufacture is energy-intensive, reducing fertilizer use has mixed effects. Energy input per hectare is reduced, but so is yield, thus increasing the relative input of cultivation energy per tonne. Reducing yield also implies a need to displace production elsewhere in order to maintain supply. This could be in areas that are less suitable and/or lead to LUC, e.g. conversion of grassland to arable, with the consequent loss of soil carbon (C). It does appear, however, that some reduction in N supply can reduce energy use per tonne bread wheat (figure 6). However, a very large reduction in N application can cause sufficient yield loss that cultivation becomes the dominant energy demand and energy use per tonne increases again.

#### (ii) Animal production

The energies used per tonne of the main outputs of animal production are all substantially higher than crops (table 2). This results from the concentration effect as animals are fed on crops and concentrate these into high-quality protein and other nutrients. Feed is the dominant term in energy use (average of about 75%), whether as concentrates, conserved forage or grazed grass. Direct energy use includes managing extensive stock, space heating for young birds and piglets, and ventilation for pigs and poultry. Housing makes up a relatively small fraction of total energy inputs, and is even lower for more extensive systems, like free-range hens. For egg production, the energy demand of manure management is more than offset by the value of chicken manure as a fertilizer, hence the negative value.

**Table 2.** Energy used in animal production at the commodity level in England and Wales. ‘ecw’ = edible carcass weight (killing out percentage * live-weight), but the energy used in slaughter is not included. 1 m^3^ milk weighs almost exactly 1 t and 15 900 eggs weigh 1 t. Source: derived from Cranfield LCA model. Williams *et al*. (2006).

commodity	poultry	pig meat	beef	lamb meat	milk	eggs
unit	1 t ecw	1 t ecw	1 t ecw	1 t ecw	m^3^	1 t
primary energy, GJ	17	23	30	22	2.7	12
feed (%)	71	69	88	88	71	89
manure & litter (%)	2	1	1	1	0	−4
housing (%)	1	4	0	0	3	3
direct energy (%)	25	26	11	11	26	12

The energy carriers used in animal production vary less than crops (table 3). About one-third is from crude oil and another third from natural gas. However, because animal feed production and supply requires 70–90% of the total energy inputs for livestock production, animal husbandry may be more vulnerable to high and volatile energy costs compared with the direct supply of arable crops. This could lead to increased pressure on extensive grazing, reversing the trends over the recent decades of decreasing land area requirements per kilogram livestock production.

**Table 3.** Energy carriers used in animal production.

	poultry (%)	pig meat (%)	beef (%)	sheep meat (%)	milk (%)	eggs (%)
crude oil	44	36	33	38	32	41
natural gas	27	28	45	46	40	28
coal	13	17	9	7	13	15
nuclear	12	15	9	7	13	12
renewable	3	3	3	2	2	4

## 3. Current ghg emissions

Agriculture occupies more than 50 per cent of the world's vegetated land (Foley *et al*. 2005) and accounts for around 20 per cent of all anthropogenic GHG emissions, depending on where the boundaries are drawn between agriculture and the other sectors, and revisions to the global warming factors assigned to each GHG (IPCC 2001, 2006; International Fertilizer Industry Association 2009). However, its contribution to methane and nitrous oxide production is disproportionately large. On a global scale, agricultural processes are estimated to account for 50 per cent of anthropogenic methane production and 80 per cent of anthropogenic nitrous oxide production (Olesen *et al*. 2006; Crutzen *et al*. 2008). As in industry, at all production stages fossil fuel combustion for heat and energy represents a direct and major source of agricultural GHG emissions. In addition, anaerobic fermentation and microbial processes in soil and manure lead to releases of methane and nitrous oxide in both livestock and arable systems. Nitrogen fertilizer production alone consumes about 5 per cent of the global natural gas supplies and significant amounts of nitrous oxide are emitted during the production of nitrate (Jenssen & Kongshaug 2003; Kindred *et al*. 2008; International Fertilizer Industry Association 2009). Furthermore, emissions as a result of LUC (mainly as carbon dioxide) can form a significant part of the agricultural impact on the atmosphere.

### (a) Arable sources

The period between 1965 and 2000 saw a doubling of global agricultural production (Tilman 1999). The total area under cultivation has remained relatively static and this huge increase in output is primarily the result of massive increases in fertilization and irrigation (figure 2; IPCC 2001), as well as improved crop genetics. Global nitrogen fertilizer applications have increased more than sixfold over the past 40 years (Tilman 1999), although there has been considerable regional variation. The production of mineral and synthetic fertilizers, especially nitrogen using the Haber–Bosch Process, uses large amounts of fossil energy, mainly natural gas, releasing around 465 Tg carbon dioxide into the atmosphere each year (International Fertilizer Industry Association 2009). It has been estimated that 30 per cent of the total fossil energy used in maize production is accounted for by nitrogen fertilizer production (Tilman 1999) and that fertilizer production is responsible for up to 1.2 per cent of all anthropogenic GHG emissions (Wood & Cowie 2004).

Fertilizer application can also lead to further emissions. Nitrification and de-nitrification of mineral and organic nitrogen fertilizers leads to the release of large amounts of nitrous oxide from soils (Snyder *et al*. 2009). The IPCC (2006) tier 1 estimate is that 1 per cent of all applied nitrogen is emitted in the form of nitrous oxide, although there is considerable uncertainty over this figure. Loss of nitrous oxide from arable soils accounts for around 1.5 per cent of total anthropogenic GHG emissions (International Fertilizer Industry Association 2009). Modern techniques that reduce soil compaction, such as GPS-guided controlled traffic farming, can reduce nitrous oxide emissions by between 20 and 50 per cent (Vermeulen & Mosquera 2009).

Emissions vary according to cultivation technique and crop type. Anaerobic turnover in rice paddies is a major source of methane (Olesen *et al*. 2006), although the anoxic conditions, when paddies are flooded minimize carbon dioxide release. Ploughing soils encourages microbial digestion of soil organic matter (SOM), leading to greater net carbon dioxide emissions. Energy use at all stages of arable production represents another significant source of carbon dioxide. However, differences in farming techniques, levels of mechanization, scales of production and soil and weather conditions in different regions make it difficult to quantify total fossil energy use and to extrapolate data from one agricultural system to another.

### (b) Livestock sources

Meat, egg and milk production are estimated to account for half of all the GHG emissions associated with food production and represent about 18 per cent of global anthropogenic emissions (Garnett 2009). In the UK, livestock farming generates 57.5 Tg carbon dioxide equivalent, which is around 8 per cent of total UK emissions (Garnett 2009). Global demand for meat and dairy products is predicted to increase over the next 50 years owing to human population growth and increased wealth. An important source of GHGs in livestock farming is enteric fermentation in ruminants, such as sheep and cattle, which produces significant quantities of methane (Olesen *et al*. 2006).

Growth of crops to feed livestock is another major source of GHG emissions. Around 37 per cent of global cereal production and 34 per cent of arable land is used to provide animal feed (FAO 2006), and so meat, egg and milk production also contributes to the release of nitrous oxide and other gases as described above. A further consideration is the efficiency with which animal feed is converted to meat. A large proportion of animal feed is respired or accumulates in non-edible parts of the animal. In the case of cattle, up to 10 kg of cereal may be required per kilogram of meat produced and so cattle farming can represent a significant demand for land and resources (Garnett 2009). Substantial differences exist between the different forms of livestock production in terms of net energy and protein feed requirements per kilogram meat produced. Increasing and volatile fossil fuel prices, unless mitigated, could drive both reductions in meat demand owing to increased prices, but also switching to the lower energy intensity, higher efficiency, forms of meat production, possibly favouring mono-gastric rather than ruminant supply chains.

### (c) Indirect emissions

On a global scale, 75 per cent of anthropogenic GHG emissions are the result of fossil fuel combustion. The remaining 25 per cent are primarily the result of LUC (Le Quéré 2009; Snyder *et al*. 2009). However, land also continues to be a net sink for carbon, absorbing about 29 per cent of total emissions, with the oceans taking up a further 26 per cent. The balance, about 45 per cent, accrues to the atmosphere (Le Quéré 2009).

Deforestation involves the removal of large above-ground biomass stocks, which represented an important carbon sink during the twentieth century (Bondeau *et al*. 2007). Below-ground biomass is lost as woody root systems and replaced by the smaller, finer roots of grasses and crop plants. Disturbance during cultivation breaks down SOM and accelerates decomposition, leading to further losses of soil carbon and, consequently, carbon dioxide emissions (IPCC 2006). The soil organic carbon content of temperate arable, grassland and woodland soils are of the order of 80, 100 and 130 t C ha^−1^, respectively (Bradley *et al*. 2005). It is thought that between 50 and 100 years are required for soil carbon content to reach a new equilibrium following LUC (Falloon *et al*. 2004; King *et al*. 2005), and so this form of disturbance leads to a long-term source of carbon dioxide. It is generally assumed that there is little difference in soil carbon between annual and perennial food crops, including fruit orchards and plantation crops (IPCC 2006). However, detailed information is lacking and further research is needed to determine the real effects of perennial crops on emissions from soils.

Deforestation in the Brazilian Amazon basin to provide land for cattle ranching and soya bean cultivation for animal feed accounts for a loss of 19 400 km^2^ of rainforest each year. This alone accounts for 2 per cent of global anthropogenic GHG emissions. While complex interlinkages and causality chains exist as drivers for deforestation, much of the soya bean grown in Brazil is exported for use as animal feed in Europe, Asia, the US and Russia. Soya bean expansion is more closely associated with Amazonian deforestation than the expansion of other crops (Volpi 2010). Overall, 7 per cent of anthropogenic emissions, totalling 2.4 Pg of carbon dioxide per year, are estimated to be the result of livestock-induced LUC (Garnett 2009). Consequently, livestock farming is a major cause of LUC. Use of former forest land for cattle ranching represents a direct LUC; use of the land to grow feed for livestock overseas represents a major indirect LUC. Each process results in further GHG emissions.

## 4. Has agricultural productivity been affected by changes in energy prices?

Fossil energy prices directly affect the costs of tillage and fertilizers and indirectly affect almost all aspects of agricultural production, through to the prices of food seen by the end consumer. The previous sections of this paper have outlined the different energy inputs and GHG emissions (energy and non-energy related) of a range of agricultural production pathways for the major food commodities. The results strongly suggest that the production costs of some agricultural commodities will be more sensitive to changing fossil fuel prices than others and that the options for mitigating the risks of fossil energy prices will also differ between those chains. This section assesses the trends in the price of oil, natural gas and coal over the last four decades and uses differences between projections for future oil prices to 2030 as a proxy for overall fossil fuel price volatility in this period.

### (a) Historic changes in fossil energy prices

**Figure 7.**
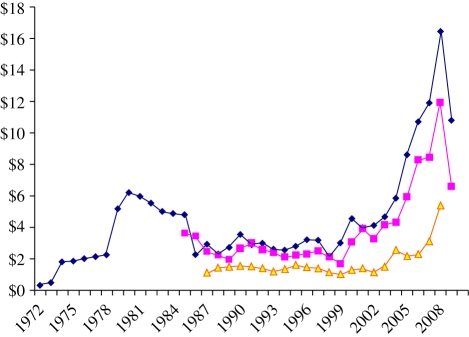
Trends in global oil, gas and coal spot-market prices; 1961–2009 (US$ per GJ). Source: BP (2009); IEA (2009). Dark blue with diamonds, oil (Dubai): $ GJ^−1^; pink with squares, gas (EU): $ GJ^−1^; yellow with triangle, coal (EU): $ GJ^−1^.

Historic trends in the spot prices of oil, natural gas and coal show that throughout the 1980s and most of the 1990s, spot prices remained below US$4 per GJ, with coal staying below US$2 per GJ until the turn of the millennium (figure 7). In fact, until 1995 fossil fuel prices were converging around US$2 per GJ, making electricity production, in particular, more attractive from natural gas than from coal because of the greater flexibility, decreased capital costs and modularity of natural gas-fired power stations. Since 1995, prices have increased first for oil then for gas and finally followed by coal. By 2007, prices for oil and natural gas had more than quadrupled, while for coal they had nearly trebled. Since then, as a result of recession and also from increased investment in new supply and refining capacity, prices have fallen sharply but more recently, since the beginning of 2009, have started increasing again, particularly for oil, although not yet to the levels seen in 2007 (BP 2009; IEA 2009; US EIA 2009).

In part, increasing supplies are a result of the deployment of new technologies, allowing hitherto inaccessible fossil fuel resources such as oil shale, tar sands or ‘tight’ gas reserves to be exploited. It is also a result of conventional supplies becoming constrained and the resulting increase in prices making previously too expensive reserves possible to access profitably. As shown in (figure 5), all agricultural commodities in the UK simultaneously use all forms of fossil-derived energy and some renewables too. A major question remains as to whether increasing overall prices and increasing volatility in those prices will drive further diversity in energy supply resources, or reductions in overall energy intensity, or even in the total supply of agricultural products.

### (b) Projected fossil energy prices

**Figure 8.**
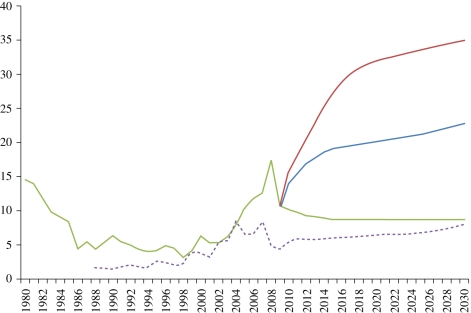
Projected oil and gas price ranges to 2030; US$ per GJ. Source: US EIA (2009). Dark blue line, reference case ($130 per bbl oil); red line, high price ($200 per bbl oil); green line, low price ($50 per bbl oil); dashed violet line, gas: 2008 US$ GJ.

As a result of real and perceived constraints to conventional fossil fuel supplies, in particular oil and natural gas, robust predictions for prices more than a few years forward are not available and the uncertainties associated with projections to 2030 are so great that the US Energy Information Administration currently uses three scenarios for oil price projections that range from US$50 to US$200 per barrel (figure 8).

For natural gas, the dominant energy feedstock for nitrogen fertilizer production, the recent development of new drilling techniques has released very substantial quantities of so-called ‘tight’ or ‘shale’ gas, reducing the price of natural gas in the US from around US$13 per MBTU in 2008 to less than US$5 per MBTU in early 2010 (The Economist 2010) or from US$12.7–US$4.3 per GJ. If tight gas is found elsewhere in substantial volumes, as seems possible, then the historic link between oil and gas prices will be broken, with oil prices likely to increase significantly and gas remaining competitive with coal.

If bioenergy, particularly biodiesel and biogas, becomes cheaper than the direct fossil fuel inputs into agriculture, primarily diesel, then a rapid switch to on-farm bioenergy is likely to occur where rotary power, transport and thermal processing are required. While the complexity of the interactions between conventional agricultural feedstocks for food and their use for energy, when coupled to global oil markets, makes this price threshold difficult to estimate, it is likely to be around US$ 70–100 per barrel oil equivalent but may be lower for large-scale commercial production facilities.

Whether this switch to bioenergy production is competitive or synergistic with food production will mainly depend on: the strength of the linkage between energy and food prices; the rate of increase of demand for bioenergy feedstocks as commodity crops; the impact from increased investment from bioenergy and the resultant increase in yields of both conventional crops (food and fuel) and advanced lignocellulosic crops; and, the availability of new land or recovered degraded or abandoned land.

## 5. Policies to reduce ghg emissions from the food sector

The impact of climate change on agricultural production is still uncertain. However, reports of the potential outcomes for agriculture are well documented (AEA 2007). Farmers in general face the looming spectre of climate change at two levels; firstly, by having to adapt existing practices to cope with the outcomes of climate change (i.e. changing weather patterns; water availability; changing patterns of pests, disease and thermal stress in livestock) and secondly, by addressing those farming activities that are contributing factors to increased GHG emissions.

While it is likely that farmers will readily adopt measures that will benefit their productivity and financial outcomes, adopting practices at a cost to farming businesses is more likely to require policy intervention. Developing mechanisms to improve GHG abatement in the agricultural sector is complex, not least because policy mechanisms are often devised through different departmental policy-making regimes.

Within the EU Climate and Energy Package (2008), the agricultural industry is not part of one of the main components, the European Emissions Trading Scheme (EU ETS 2009). Agriculture, as a non-EU ETS sector, is charged with reducing emissions to 10 per cent below 2005 levels by 2020, and it is anticipated that this will be through binding national targets. In the policy context, the farming industry faces many challenges before carbon trading as an economic strategy becomes a reality.

The UK Government published its low carbon transition plan in 2009 (http://www.theccc.org.uk/carbon-budgets). The Plan's main points for *agriculture* are to:

— Encourage English farmers to take action themselves to reduce emissions to at least 6 per cent lower than currently predicted by 2020, through more efficient use of fertilizer and better management of livestock and manure;
— Review voluntary progress in 2012, to decide whether further government intervention is necessary. The Government will publish options for such intervention in Spring 2010;
— Ensure comprehensive advice programmes are available to support farmers in achieving this aim, to reduce their emissions from energy use, and to save money in the process;
— Research better ways of measuring, reporting and verifying agricultural emissions;
— Encourage private funding for woodland creation to increase forest carbon uptake;
— Provide support for anaerobic digestion, a technology that turns waste and manure into renewable energy via biogas; and
— Reduce the amount of waste sent to landfills, and better capture of landfill emissions.

Some policy instruments that aim to deliver GHG mitigation within the sector have been identified in a report commissioned by the UK's Department for Food and Rural Affairs (ADAS 2009). The report shows the mitigation potential by 2022 (table 4), making comparisons to an earlier Scottish Agricultural College report (SAC 2008). The study does not include mitigation potential from biomass production, soil carbon sequestration or options for anaerobic digestion of farmyard waste, and does not expand on further economic or market-based policy mechanisms (e.g. carbon trading extending to farming activities). The policy instruments identified are as follows:

— Regulatory—Cross compliance and nitrate pollution prevention regulations (nitrogen vulnerable zone (NVZ) regulations);
— Economic (voluntary participation)—environmental stewardship; and
— Voluntary—extend catchment sensitive farming (CSF), farm assurance public procurement, voluntary agreements and targeted communications.

**Table 4.** Scale of UK agricultural abatement potential by 2022 by policy instrument (ktCO_2_e per year; ADAS 2009).

policy	SAC	ADAS
extend coverage of NVZs to 100% farmed area	not covered	
extend area and scope of NVZs	2531	602
targeted communications	351	212
voluntary agreements	480	238
farm assurance public procurement	10	6
cross-compliance—additional standards within existing rules	896	896
cross-compliance—extend scope through negotiations with EU	3420	1491
environmental stewardship	647	647
enhance CSF—to 100% farmed area	515	200
enhance CSF—extend area and scope	648	333

### (a) Indirect policy implications for agricultural emissions

Policies to reduce emissions from the fossil energy sector may impact on agriculture in two different ways. Firstly, by promoting crops that can be used as feedstocks for biofuel or bioenergy; different growing regimes and more efficient energy inputs may be adopted. Secondly, GHG emission reporting requirements that are being developed for biofuels may affect farming practices, particularly if benefits for improved emissions are transferred down the supply chain to the feedstock producers. Policies in the UK that aim to impact fossil fuel energy use and, which in turn may impact on agriculture are the renewable transport fuels obligation (RTFO; DfT 2007) and the renewables obligation (RO; DTI 2007).

In the EU, the climate and energy package (2008) committed the 27 member states to reduce CO_2_ emissions by 20 per cent, and to target a 20 per cent share of energy supply from renewable energy by 2020 i.e. the so-called ‘20–20 in 2020’. Policy instruments in the package, which may then indirectly impact on agriculture, are the Fuels Quality Directive (EU FQD 2009) and the Renewable Energy Directive (EU RED 2009). The FQD aims to reduce harmful atmospheric emissions, including GHGs, and includes mandatory monitoring of life cycle GHG emissions. The RED aims to promote renewable energies and has a component that addresses sustainability of biofuels and the land used to grow biofuel feedstocks.

In the United States, the California Environmental Protection Agency Air Resources Board (CARB) has been at the forefront of developing policy to reduce emissions from fossil energy and has developed the low carbon fuels standard (LCFS 2007). This standard is under review by a number of individual states in the US, which are also looking to adopt an emissions approach to the inclusion of biofuels in transport fuels. Nationwide in the US, the Environmental Protection Agency (EPA) has developed, under the Energy Independence and Security Act of 2007, a renewable fuel standard programme (RFS2 2009) that aims to increase the volume of renewable fuel in gasoline from 9 billion gallons (34 billion litres) in 2008 to 36 billion gallons (144 billion litres) by 2022.

In many ways, these policies are leading the development of methodologies that will improve energy efficiency and reduce GHG emissions across supply chains. Improving emissions and ensuring the sustainability of biofuels have led to the development of variety of policy-specific methodologies. They have also encouraged the formation of global stakeholder interactions, which address environmental, economic and social issues e.g. Roundtable on Sustainable Biofuel (RSB); Global Bioenergy Partnership (GBEP) and crop-specific initiatives e.g. Roundtable on Sustainable Palm Oil (RSPO), Round Table on Responsible Soy (RTRS) and the Better Sugar Cane Initiative (BSI).

The UK's RTFO has been devised with GHG emissions monitoring and reduction as a key component and it has been necessary to stipulate methodology and processes to report GHG emissions from the individual biofuel supply chains used by obligated parties in law (RFA 2009). The RTFO's carbon and sustainability methodologies cover biofuel supply chains from feedstock source, by country and by on-farm production inputs and outputs. In a biofuel supply chain, this may encourage farmers to improve management practices, providing that a share of the value or benefits feed back to farmers. Currently, carbon and sustainability reporting is not mandatory under the RTFO and better practices leading to improved carbon and sustainability profiles are not rewarded. Many farmers in the UK have been encouraged by the idea of reducing on-farm diesel costs by producing their own biodiesel from oilseed rape. However, the market value of vegetable oil and costs for processing oils into biodiesel will always be calculated against fossil diesel costs for farm use (Lewis 2009). Furthermore, farm vehicles will generally be under warranty from the vehicle manufacturer and it is unlikely that farmers would risk using out-of-spec fuel, to the detriment of these costly machines.

As noted by Monbiot (2009), addressing energy needs using on-site, renewable energy options only reduces dependence on diesel for on-farm use by a quarter. Options for farmers to use renewable energies, such as biomass or biogas for electricity and heat production, are often limited to on-farm use only, as there are not the facilities or incentives to connect to the electrical grid. Allowing access to the national grid would give farmers an option to trade renewable energy under the RO, whereby the mandatory renewable requirement of 15 per cent electricity by 2015 could potentially be met in part by surplus on-farm energy generation, traded as renewable energy certificates (ROCs). The UK Government is also reviewing opportunities for a renewable heat incentive (RHI), under the Energy Act (DECC 2008), which promotes investment for biomass boilers and combined heat and power (CHP) facilities.

## 6. Options for agriculture to reduce its dependence on energy

### (a) Change tillage / pre-processing

Land preparation has become increasingly mechanized over the years. However, mechanical tillage systems are energy-intensive and expose SOM to decomposition, leading to enhanced GHG emissions, reduced SOM concentration in soil and, potentially, in the short and longer term, to soil erosion and degradation. The potential for reducing the energy intensity of agricultural production by adopting alternative tillage systems may occur from decreased fuel use in mechanical operations or as the result of better long-term soil productivity.

Alternative methods of land preparation and crop establishment have been devised to reduce energy requirements and maintain good soil structure. These include minimum tillage (min-till), conservation tillage (no tillage or min-till) and direct drilling resulting in increased surface organic matter from previous crops residues (soil coverage of 30%; Van Den Bossche *et al*. 2009). Robertson *et al*. (2000) compared management techniques in a three-crop rotation over 8 years in Michigan. The net changes in soil C (g m^−2^ yr^−1^) were for conventional tillage (plough-based tillage), 0; organic with legume cover, 8.0; low input with legume, 11 and no till, 30.

The consequences of reduced tillage on soil carbon are not straightforward. Baker *et al*. (2007), concluded that the widespread view that reduced tillage favours carbon sequestration may be an artefact of sampling methodology, with reduced tillage resulting in a concentration of SOM in the upper soil layer rather than a net increase throughout the soil. They did, however, highlight that there were several good reasons for implementing reduced tillage practices. In contrast to Baker *et al*. (2007), Dawson & Smith (2007) reviewed the subject area and suggested sequestration rates of 0.2 (0–0.2) and 0.39 (0–0.4) t C ha yr^−1^ for reduced tillage and no-till farming, respectively.

Energy balance calculations resulting from fertilizer application are more difficult to assess, as interactions with increased SOM become more complex. Studies that focus on energy inputs, attributed to soil preparation, tend to be regional and crop-specific. Energy from tillage will depend on crop requirements, soil type, cultivation/climatic conditions, equipment used and engine efficiency.

**Figure 9.**
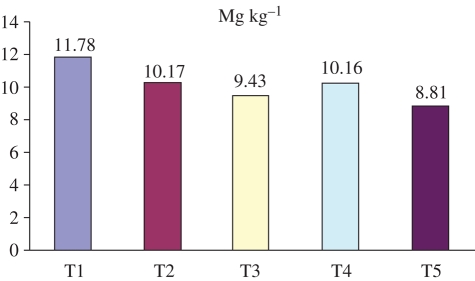
Energy consumed for 1 Kg wheat production in Maragheh region of Iran. Source: Tabatabaeefar *et al*. (2009). T1, mold board plough + roller + drill; T2, chisel + roller + drill; T3, cyclo-tiller + drill; T4, sweep + roller + drill; T5, no-till + drill.

A study that compares conventional and integrated farming in the UK attributed energy savings in integrated farming almost entirely to the reduction in energy required for mechanical operations (Bailey *et al*. 2003). The study also considered the effects on energy of multi-functional crop rotation, integrated nutrient and crop protection methods, and ecological infrastructure management (i.e. field/farm boundary maintenance to promote biodiversity and reduce pollution), in integrated systems. A study for wheat grown in Iran provides a more detailed evaluation of five specific tillage regimes (Tabatabaeefar *et al*. 2009). The study reports the min-till system (‘T5’ in figure 9) as the most energy-efficient, with energy for tillage accounting for 19 per cent of the total energy versus 32.5 per cent for the least energy-efficient (‘T1’). Yield outcomes are also reported whereby the min-till system gives the second-highest yield of the five systems, but in overall performance ‘T3’ is reported as being the most efficient system when taking both energy input and yield into account.

Soil carbon as a component of SOM is important in carbon turnover within the carbon cycle, and in maintaining soil fertility, water and nutrient-holding capacity, ecosystems functions and preventing soil degradation. Soil carbon and SOM are important in preserving soil in a productive, quality state for long-term crop production (Dawson & Smith 2007). Understanding the processes of carbon interaction in soils is complex, both at local and national levels. Carbon losses from the SOM pool, the effect of carbon loss on nutrient availability and crop productivity, and the subsequent outcomes for agricultural management activities are all important variables in calculating the overall carbon stocks and productivity of soils (Dawson & Smith 2007). Other farming options, such as residue mulching and the use of cover crops, aim to conserve and enhance SOM or soil carbon sequestration (Lal 2007).

The subsequent effects of nutrient availability on crop productivity vary between cropping systems (e.g. conventional or organic systems), land types, climatic conditions and time, and require further research before being fully integrated into farming systems (Kong *et al*. 2009). Studies carried out on sites in Belgium have been used to demonstrate nitrogen interactions under various planting regimes and to demonstrate the action of tillage on organic matter degradation and the subsequent availability of nitrogen in the nutrient pool over time (Van den Bossche *et al*. 2009). They report higher SOM, microbial biomass and enzymatic activity for conservation tillage, which increases with time. The anticipated effect is slower mineralization or immobilization of nitrogen, leading to enhanced soil fertility as the result of long-term build-up of nutrient reserves of the soil.

Understanding the interaction between soil carbon and nitrogen also adds further complexity to determining the benefits of increasing soil carbon through changes in tillage systems. While increasing fertilizer inputs may increase the soil carbon pool, the poorer GHG balance from the increased use of nitrogen fertilizers may negate the sequestration benefit. The reasons for changing agricultural activities should be clear from the outset. Is the anticipated benefit to reduce energy inputs, reduce GHG emissions, improve soil carbon sequestration or to maintain the long-term productivity of soils? Land management choices may then follow, with trade-offs expected and accepted—for example, planting marginal lands with biomass crops to improve carbon sequestration versus maximizing yields on productive lands by increasing fertilizer use, or adopting min-till systems on land areas where mechanical activities are also degrading soil quality or causing soil erosion, such as on sloping sites.

### (b) Energy inputs and impacts of fertilizer use in agriculture

**Figure 10.**
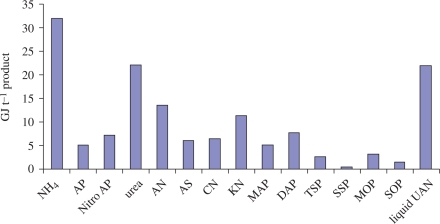
Energy inputs into the main fertilizer building blocks; European average technology. Source: Jenssen & Kongshaug (2003).

In addition to the direct energy inputs for tillage and harvesting, fertilizers can constitute a significant share of total energy inputs to agriculture (figure 4) and food production, particularly for nitrogen-intensive crops such as cereals. Figure 10 shows the different energy requirements for the main constituents of commercial fertilizers, using European average technologies. The main nitrogen components of fertilizers, ammonia (NH_4_; 32 GJ t^−1^), urea (22 GJ t^−1^) and liquid UAN (urea AN; 22 GJ t^−1^), are the most energy-intensive to produce, while the P and K components all require less than 5 GJ t^−1^ to produce.

The energy inputs needed to produce and supply fertilizers and pesticides substantially outweigh the energy required to apply the products in the field. GHG emission factors for production, supply and use of N, P and K fertilizers, under average UK conditions, are provided in table 5. However, for nitrogen fertilizers, the GHG emissions arise both as a result of the fossil energy inputs needed to capture and process atmospheric nitrogen, and also from complex soil-based processes that result in the production and release to the atmosphere of nitrous oxide (N_2_O) in-field.

**Table 5.** GHG emission factors for fertilizers, seeds and pesticides. Source: Woods *et al*. (2008).

agricultural input	GHG emissions (kg CO_2_eq kg^−1^ applied)
nitrogen fertilizer (as N)	6.69
phosphate fertilizer (as P)	0.71
potash fertilizer (as K)	0.46
lime	1.80
pesticides (as active ingredient)	5.41
seed material	0.87

#### (i) Nitrogen fertilizers

**Figure 11.**
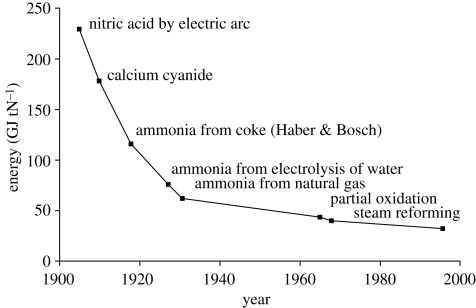
Historic development in energy requirements in N-fixation for nitrogen fertilizer. Source: Konshaug (1998).

The energy inputs into nitrogen fertilizer production have decreased significantly since the beginning of the last century as a result of continual technological innovation (figure 11). GHGs emitted during its production include carbon dioxide, methane and nitrous oxide as shown in table 6. Carbon dioxide emissions account for 98 per cent of the GHG emissions on a mass basis, but only 33 per cent on a global warming potential (CO_2_ equivalent) basis. N_2_O accounts for 0.6 per cent of the mass of the GHG released but 65 per cent on a CO_2_ equivalent global warming potential basis.

**Table 6.** Primary energy inputs and greenhouse gas emissions associated with ammonium nitrate manufacture in Europe. Source: Elsayed *et al*. (2007).

nitrogen fertilizer manufacture	primary energy inputs (MJ kg N^−1^)	carbon dioxide emissions (kg CO_2_ kg N^−1^)	methane emissions (kg CH_4_ kg N^−1^)	nitrous oxide emissions (kg N_2_O kg N^−1^)	total greenhouse gas emissions (kg kg N^−1^)
ammonium nitrate	40.74 ± 5.43	2.30 ± 0.26	0.012 ± 0.001	0.015	2.33
kg CO_2_ eq kg N^−1^		2.30	0.28	4.44	6.93 ± 0.26

However, while ammonia production is the most energy-intensive part of the production of nitrogen fertilizers, nitric acid production causes the release of N_2_O during its production. Nitric acid is needed to produce AN through a reaction with ammonia. The N_2_O leaks to the atmosphere in the nitric acid plants and between 70 and 90 per cent of this N_2_O can be captured and catalytically destroyed. European plants are now being fitted with this nitrous oxide abatement technology and as a result overall AN GHG emissions could be reduced, by 40 per cent overall, from 6.93 to 4.16 kg CO_2_ eq kg N^−1^.

### (c) Farm forestry systems *(*agro-forestry*)*

**Figure 12.**
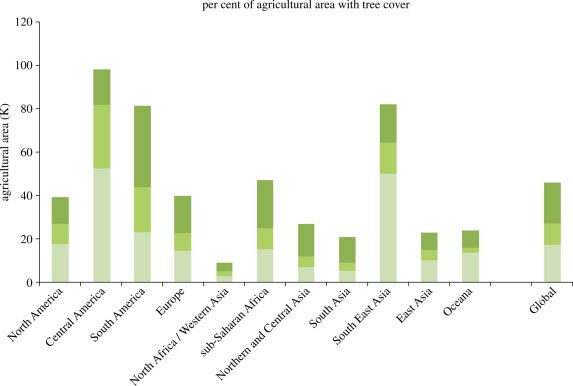
Percentage of world agricultural land that can be regarded as being under agro-forestry systems to varying intensities. Source: after Zomer *et al.* (2009). Dark green bars, >10%; green bars, >20%; light green bars >30%.

The production of woody biomass on land unsuitable for intensive arable farming or extensive grazing is widely seen as a low-energy input option, for the production of such biomass for material or energy usage. Numerous opportunities exist to integrate the production of woody biomass and agricultural crops or livestock and production and such ‘farm-forestry’ or ‘agro-forestry’ systems have been widely discussed in the literature and through the work of the consultative group on International Agricultural Research's (CIGIAR) World Agroforestry Centers,^1^ much of which is focused on the developing world. A recent geospatial study by Zomer *et al*. (2009) has shown agro-forestry to be a significant feature of agriculture in all regions of the world (figure 12).

Zomer *et al*. (2009) provide a cautious estimate that 17 per cent (approx. 3.8 million km^2^) of global agricultural land involves agro-forestry at greater than 30 per cent tree cover and, potentially, this can be as high as 46 per cent or just over 10 million km^2^, at greater than 10 per cent or more tree coverage rates. Agro-forestry systems are found in developed as well as less-developed regions.

The widespread and significant proportion of agricultural land under agro-forestry management (e.g. in Central and South America) already points to a successful form of integrated land management for both crop production and woody biomass for energy production. This indicates a capacity for agricultural land management to accommodate integrated energy production; currently, in most cases, the woody biomass is used for immediate local needs such as fuelwood for cooking. However, there is also considerable scope for more widespread introduction of tree or coppice material to agricultural land specifically to meet on-farm energy needs and, subject to transportation constraints, as an economic product for off-farm sale. For example, in the UK, a number of estates are currently using wood produced on the estate for biomass heat schemes, which is encouraged under the UK's Bioenergy Capital Grant Scheme.

With combinations of increasing prices for conventional energy inputs to farming and incentives for low-carbon forms of renewable energy, farmers may be incentivized to allocate a proportion of their crop land to meet on-farm energy use, for example, for diesel fuel replacement or potentially for high-value low-carbon certified electricity, either produced on-farm or from farm-derived woody/residual feedstocks. The ability to co-produce woody biomass for heat and/or power generation at farm scale, alongside commodity crops, provides a potentially attractive route to mitigating increased or volatile external energy costs (e.g. for drying, livestock management or domestic use) and potentially as a saleable commodity in its own right (biomass fuel product(s)).

Future incentivization for farmers to minimize agricultural GHG emissions is also likely to favour greater integration of forestry and/or woody biomass cultivation on-farm e.g. short rotation coppice or perennial grasses such as *Miscanthus* in UK/EU. At the individual farm level, cultivation of perennial biomass crops on a proportion of the land may provide an attractive route to ‘balance’ more GHG-intensive cultivation activities with carbon ‘credits’ from enhanced C-storage in soils, via avoided emissions from displaced fossil fuel requirements or as a direct economic benefit from biomass sales at a premium owing to renewable heat and power incentive value trickling down the supply chain. Recent studies by Hillier *et al*. (2009) have illustrated the GHG benefits associated with soil carbon storage effects for certain biomass crops and land-use transition scenarios modelled in a LCA context for England and Wales. Attention is also being given to the use of biochar^2^ as a potential energy source (during the charring process) and significantly as a soil-based carbon sequestration and storage approach that can also offer soil fertility benefits (Collison *et al*. 2009; Sohi *et al*. 2009). Biomass supply for biochar production can be drawn from diverse sources, including woody biomass from agro-forestry systems as well as from existing UK farm biomass, such as hedgerow management (A. Gathorne-Hardy 2009, personal communication).

## 7. Concluding remarks

This paper has identified that there are significant risks to future farming and yields owing to increasing and increasingly volatile fossil fuel prices. While it has been difficult to obtain robust projections for oil, natural gas and coal prices, it is clear that:

— Fossil fuel prices, particularly those of oil-derived products, will increase significantly over the coming decades and will become more volatile.
— Prices, on a unit energy basis, between oil, gas and coal, are likely to diverge with the possibility of a break in the traditional linkage between gas and oil prices emerging. Unless substantive agreements emerge from the UNFCCC's inter-governmental negotiations that limit access to coal, its large and widely distributed reserves will mean that it is the least vulnerable of the fossil fuels to price increases; a switch to coal away from oil and natural gas is probably where that is possible e.g. for processing and nitrogen fertilizer production.
— The world's major crops are dependent on different shares of their energy inputs from oil, gas and coal. Thus, relative changes in fossil fuel prices will affect each crop type differentially.
— Major areas of concern are:
  - Increasing oil prices will directly affect the price of diesel used for tillage, transport of crops from fields, and from storage to processing and end use.
  - Increasing natural gas prices will have the most immediate effect on nitrogen fertilizer prices.
  - Coal is still used for nitrogen fertilizer production, particularly in China, and is likely to be least affected by worries about reserve depletion. From a GHG perspective, a switch away from oil and gas to coal, rather than to renewable, would be detrimental.
  - Increased costs for direct and indirect energy inputs into agriculture may lead to lower yields for the world's major agriculture commodity crops. In turn, this is likely to lead to an expansion of land areas under these crops, leading to increased GHG emissions, as a result of LUC, and increased prices owing to less efficient production. Significant land expansion will also have detrimental effects on biodiversity and possibly on water resources.
— Reasons for optimism
  - Substantial gains in efficiency of energy use and GHG emissions are possible in all areas of food and bioenergy supply chains and from both conventional and advanced supply chains.
  - Recent policy developments for bioenergy, and in particular, biofuels, have demonstrated that the highly complex and heterogeneous systems necessary to account, monitor, reward and penalize good or bad GHG and wider sustainability criteria, are amenable to policy. It is possible, and indeed necessary, that many of the lessons learnt in developing these policies and mechanisms for biofuels can be applied to any form of biological production including food.
  - New tools, in particular spatial zoning and land management tools, are highlighting the potential for revised management and crop choices that could allow enhanced carbon stocking and biodiversity from integrated land management and planning that couples annual and perennial agriculture.
  - The developing of novel drilling technologies that have enabled access to ‘tight’ gas reserves in the US may delay a switch to coal and reduce inflationary pressures on nitrogen fertilizer prices.

While increasing fossil fuel prices could pose a major risk to agriculture as production costs increase, and also cause increased volatility in prices between the different major agricultural commodities, there is substantial scope for technological and management innovations to occur, decreasing the dependence on fossil energy supplies and creating opportunities for new markets e.g. in renewable energy. The opportunities and threats will vary substantively between the different crops and a careful review on a crop-by-crop basis is necessary to understand and manage these threats and the risks to future production posed by increasing fossil fuel prices.

## Endnotes

1 See http://www.worldagroforestry.org/af/.

2 Biochar is carbonised biomass or charcoal. When biomass is turned into charcoal and applied to soils it is believed to have a half-life in the soil in order of 1000 years.
